# The Relationships between Chemical and Genetic Differentiation and Environmental Factors across the Distribution of *Erigeron breviscapus* (Asteraceae)

**DOI:** 10.1371/journal.pone.0074490

**Published:** 2013-11-18

**Authors:** Xiang Li, Li-yan Peng, Shu-dong Zhang, Qin-shi Zhao, Ting-shuang Yi

**Affiliations:** 1 Key laboratory of Biodiversity and Biogeography, Kunming Institute of Botany, Chinese Academy of Sciences, Kunming, China; 2 Graduate University of Chinese Academy of Sciences, Beijing, China; 3 Plant Germplasm and Genomics Center, Germplasm Bank of Wild Species, Kunming Institute of Botany, Chinese Academy of Sciences, Kunming, China; 4 State Key Laboratory of Phytochemistry and Plant Resources in West China, Kunming Institute of Botany, Chinese Academy of Sciences, Kunming, China; Centro de Investigación y de Estudios Avanzados del IPN, Mexico

## Abstract

**Aims:**

*Erigeron breviscapus* (Vant.) Hand.-Mazz. is an important, widely used Chinese herb with scutellarin, 1,5-dicaffeoylquinic acid, 3,5-dicaffeoylquinic acid and erigoster B being its major active compounds. We aimed to resolve the influence of biotic and abiotic factors on the concentrations of these compounds and to determine appropriate cultivation methods to improve the yields of the four compounds in this herb.

**Methods:**

In order to detect the major genetic and natural environmental factors affecting the yields of these four compounds, we applied AFLP markers to investigate the population genetic differentiation and HPLC to measure the concentrations of four major active compounds among 23 wild populations which were located across almost the entire distribution of this species in China. The meteorological data including annual average temperature, annual average precipitation and annual average hours of sunshine were collected. The relationships among the concentrations of four compounds and environmental factors and genetic differentiation were studied.

**Important Findings:**

Low intraspecific genetic differentiation is detected, and there is no obvious correlation between the genetic differentiation and the contents of the chemical compounds. We investigated the correlation between the concentrationsof four compounds (scutellarin, 1,5-dicaffeoylquinic acid, 3,5-dicaffeoylquinic acid and erigoster B) and environmental factors. Concentrations of two compounds (1,5-dicaffeoylquinic acid and 3,5-dicaffeoylquinic acid) were correlated with environmental factors. The concentration of 1,5-dicaffeoylquinic acid is positively correlated with latitude, and is negatively correlated with the annual average temperature. The concentration of 3,5-dicaffeoylquinic acid is positively correlated with annual average precipitation. Therefore, changing cultivation conditions may significantly improve the yields of these two compounds. We found the concentration of scutellarin positively correlated with that of erigoster B and 3,5-dicaffeoylquinic acid, respectively. We inferred that the synthesis of these two pairs of compounds may share similar triggering mechanism as they synthesized in a common pathway.

## Introduction

The genus *Erigeron* L. (Asteraceae: Tribe Astereae) includes 390 species worldwide [1]. Species of *Erigeron* are herbs or rarely subshrubs, usually perennial, rarely annual or biennial. Thirty-five *Erigeron* species are found in China (13 species are endemic to China), most of which are distributed in western China. *Erigeron breviscapus* (Vant.) Hand.-Mazz. is an important herb in traditional Chinese medicine and has been used to treat cardiovascular and cerebral vessel diseases [2]–[3]. This species is a perennial herb, which is endemic to southwestern China at elevations between 1000 m and 3500 m. It is mainly distributed in mid-altitude mountains and subalpine open slopes, grasslands and forest margins [4]. *Erigeron breviscapus* is diploid [5] and has an outcrossing mating system [6].

The chemical components of *E. breviscapus* have been subject of extensive studies for its medicinal value. A series of flavonoids and other phenols from this herb have been proven to be effective in treating cerebral infarction, digestive disorders and heart diseases [3], [7]–[10]. Among them, scutellarin, 1,5-dicaffeoylquinic acid, 3,5-dicaffeoylquinic acid and erigoster B are four major active components. Scutellarin, scutellarein 7-*O*-*β*-D-glucuronide, is one of the major flavonoid glucuronides isolated from this species. This compound can inhibit [^3^H]-LSD binding to the serotonin-7 receptor (5-HT_7_ receptor) [11], and has protective effects on cardiovascular and cerebrovascular ischemia in rats [12]. The antioxidant property of 1,5-dicaffeoylquinic acid has been confirmed to inhibit chemiluminescence [13] and methyl linoleate hydroperoxide formation [14]. 3,5-Dicaffeoylquinic acid possesses neuroprotective properties against neuronal cell death induced by oxidative stress, which may be useful for therapeutic protection as well as treatment of neurodegenerative diseases [15]. According to Sun and Zhao [16], 1,5-dicaffeoylquinic acid, 3,5-dicaffeoylquinic acid and erigoster B, the major conjugated hydroxycinnamates in *E. breviscapus*, have similar bioactivities with that of scutellarin. They have antioxidant properties, vessel-dilation activity and certain inhibitory effects on adenosine diphosphate (ADP) induced platelet aggregation in rats. Since *E. breviscapus* has profound medicinal functions, the demand for it has been increased rapidly. Consequently, finding methods to raise its yield is urgently needed, and thus we focused our study on ways to increase the concentration of scutellarin, 1,5-dicaffeoylquinic acid, 3,5-dicaffeoylquinic acid and erigoster B in *E. breviscapus*.

These four major chemical compounds are phenols, which are important plant secondary metabolites that have various functions in adapting to both micro- and macro-environments, such as water stress, temperature stress, UV light and disease resistance [17]. External stimuli can modulate their synthesis and therefore change the chemical composition or quantities of phenolic compounds (such as flavonoids and conjugated hydroxycinnamates) in plants [18]–[19]. According to de Abreu and Mazzafera [20], water stress on *Hypericum brasiliense* particularly increases the levels of phenolic compounds, and plants that are kept at a constant low or high temperature have higher levels of phenolic compounds. In *Hypericum* sp. (St. John's wort), higher temperatures can cause higher concentrations of pseudohypericin and hypericin in its shoot tissues [21]. Nevertheless, these studies have been carried out in a greenhouse environment. As to *E. breviscapus*, previous studies have been resolved the relationships between the content of phenols and water stress [22], CO_2_ concentration [23], nitrogen concentration in the soil [24], and UV light intensity [25] under control conditions. However, there are no studies to address the impact of natural environmental factors on the content of phenols in *E. breviscapus*. Since *E. breviscapus* is usually cultivated in outdoor farmlands rather than in greenhouses, investigating the linkage between natural environmental factors and the content of phenols will supply basic information for choosing optimal cultivation area and techiniques. Therefore, we gathered data for environmental factors including annual average temperature, annual average precipitation, annual average hours of sunshine from the collecting sites to detect effects of related factors on the four major compounds. Although *E. breviscapus* is perennial herb, the above-ground portion dies during autumn, and new shoots germinate in spring. The compounds of this herb are mainly isolated from newly developed shoot, which is primarily affected by environmental factors of the related growing season. We thus only included the annual average value in our analyses. The relationship between amounts of medicinal compounds and the population's geographic distribution was ignored in the previous researches on *E. breviscapus*. However, this relationship is crucial for choosing optimal cultivation locations, and thus the relationships between the main distribution information (latitude, longitude and altitude) and the four major compounds was also analyzed.

In addition to environmental factors, the composition and content of chemical compounds are closely correlated with the genetic differentiation of the related organism [26]–[28]. However, this relationship has not been previously addressed in *E. breviscapus*. We investigate the relationship between genetic and chemical differentiation of four compounds in order to reveal the potential high yield lineages. Multiple kinds of molecular markers have been applied in similar studies to resolve intraspecific genetic differentiation. Amplified fragment length polymorphisms (AFLPs) is an effective way to investigate population genetic structure and diversity and has become one of the most widely used molecular markers to study genetic differentiation since Vos [29] first published it in 1995. We chose AFLPs to investigate the intraspecific genetic differentiation of *E. breviscapus*, and we used high-performance liquid chromatography (HPLC) to measure the percentage of scutellarin, 1,5-dicaffeoylquinic acid, 3,5-dicaffeoylquinic acid and erigoster B in each population. By analyzing relationships among population genetic differentiation, contents of the four chemical compounds and the environmental factors mentioned above, the main purpose of this study was to resolve the influence of biotic and abiotic factors on the concentrations of these compounds and to seek appropriate measures to improve the yields of this crucial medicinal herb.

## Materials and Methods

### Ethics statement

All specimens were collected in locations for which specific permission was not required. None of these locations were protected areas or private lands. *Erigeron breviscapus* is not currently a protected species in China, therefore, no specific permission was required to carry out our fieldwork.

### Plant materials and environmental data sampling

**Figure 1:**
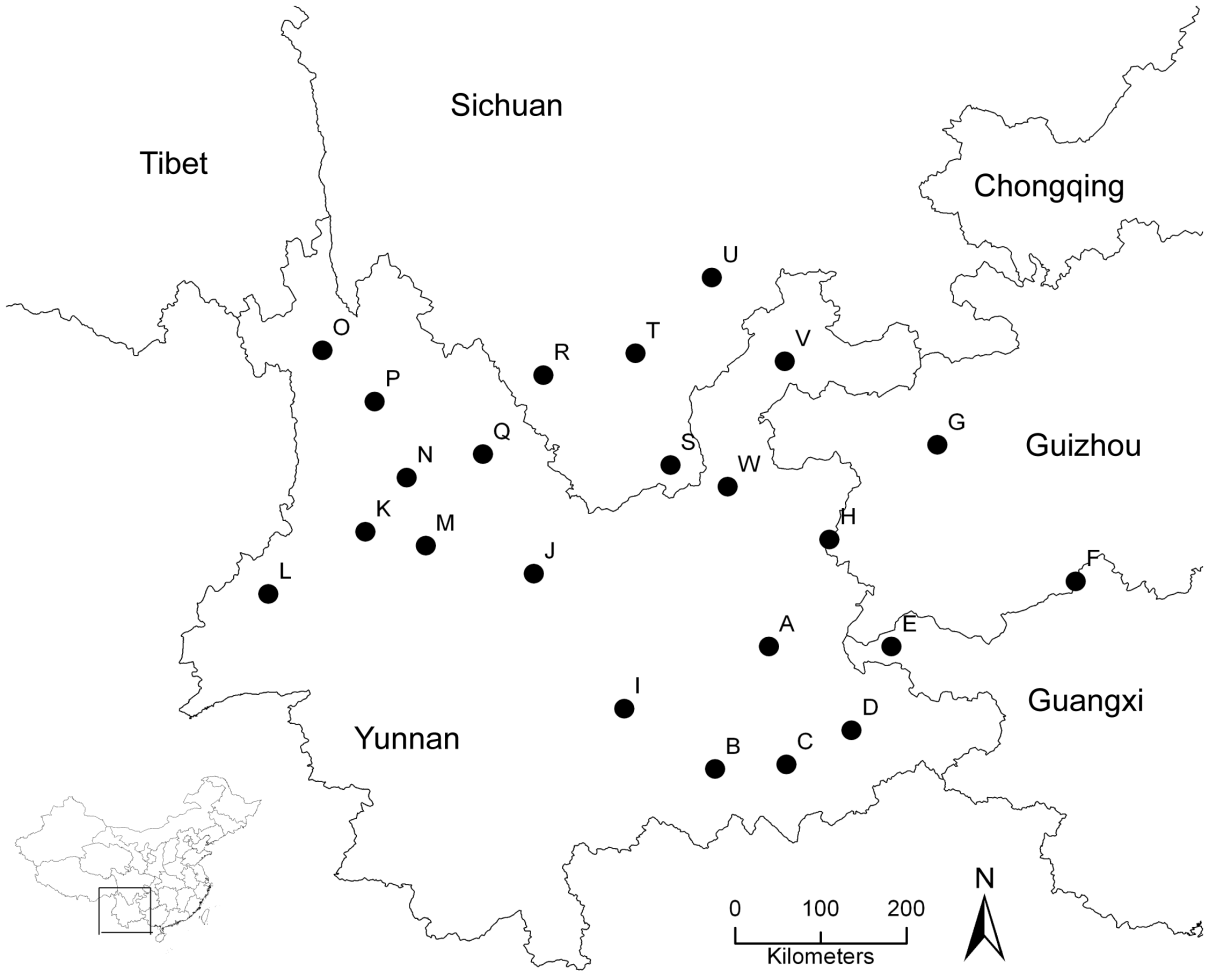
Distribution of the sampling locations (capital letters denote populations).

Twenty-three populations were sampled across the distribution of *E. breviscapus* (Fig. 1). Ten to twenty flowering individuals (whole plant) from each population were randomly collected for chemical examinations. In order to diminish seasonal effects on the content of the chemical components, all materials were collected from mid-July to mid-August in 2009. From *E. breviscapus'*s early flowering phase to its full-bloom phase, the contents of the four studied active compounds are highest (unpublished data from Liyan Peng, one coauthor of this article). For genetic analyses, 15 individuals from each population were sampled, with individuals at least 30 m apart.

The meteorological data for 19 populations including annual average temperature, annual average precipitation and annual average hours of sunshine in 2009 were obtained from an online database (http://cdc.cma.gov.cn/index.jsp) and local meteorological stations (Table 1). We failed to get meteorological data for the remaining four populations, which were omitted in the analyses between compound concentration and environmental factors. The distributional information of latitude, longitude and altitude were gathered during the field work.

**Table 1:** The sampling information for 23 *E. breviscapus* populations, including environmental data.

Population ID	Province	County	Latitude	Longitude	Altitude (m)	Annual average	Annual average	Annual average
						temperature (°C)	precipitation (mm)	hours of sunshine (h)
**A**	Yunnan	Luxi	N24°37′	E103°43′	1830	15.7	585.3	1970.7
**B**	Yunnan	Gejiu	N23°18′	E103°09′	2062	15.9	1069.7	1968.6
**C**	Yunnan	Wenshan	N23°21′	E103°54′	2560	17.8	1015	2088.9
**D**	Yunnan	Yanshan	N23°43′	E104°35′	1600	16.9	705.5	1900
**E**	Guangxi	Longlin	N24°37′	E105°00′	1550	—a	—a	—a
**F**	Guizhou	Luodian	N25°19′	E106°56′	1176	20.6	846.1	1375.4
**G**	Guizhou	Nayong	N26°47′	E105°29′	1900	—a	—a	—a
**H**	Yunnan	Fuyuan	N25°46′	E104°21′	2207	13.8	1096	1833.3
**I**	Yunnan	Xinping	N23°57′	E102°12′	2165	17.4	952.7	2252.4
**J**	Yunnan	Yaoan	N25°24′	E101°15′	2000	15.2	790.3	2500.5
**K**	Yunnan	Yunlong	N25°51′	E099°29′	2190	13.2	879	1837.7
**L**	Yunnan	Tengchong	N25°11′	E098°28′	1950	16.1	1185.1	2475.2
**M**	Yunnan	Dali	N25°42′	E100°07′	2410	16.1	1049	2396.9
**N**	Yunnan	Jianchuan	N26°26′	E099°55′	2270	12.3	776.7	2439.8
**O**	Yunnan	Weixi	N27°48′	E099°02′	1880	11.3	946.2	2110.4
**P**	Yunnan	Yulong	N27°15′	E099°35′	1950	12.6	962.3	2546
**Q**	Yunnan	Yongsheng	N26°41′	E100°43′	2155	13.5	955	2439.9
**R**	Sichuan	Yanyuan	N27°32′	E101°21′	2500	13.1	759.6	2703.3
**S**	Sichuan	Huidong	N26°34′	E102°41′	2368	—a	—a	—a
**T**	Sichuan	Xichang	N27°46′	E102°19′	1796	17.8	908.1	2183.6
**U**	Sichuan	Meigu	N28°35′	E103°07′	2313	—a	—a	—a
**V**	Yunnan	Daguan	N27°41′	E103°53′	1162	15.3	1068.6	2475.6
**W**	Yunnan	Huize	N26°20′	E103°17′	2250	14.1	623.4	2325

a Lack of meteorological data.

### High-performance liquid chromatography

In order to reduce individual variation, the individual specimens were dried out under the room temperature, and then individuals from each population were pooled together. Each dried sample was ground into powder and weighed (5 g), and extracted with aqueous acetone (50%) in an ultrasonic bath for 40 min. The extracts were purified for HPLC analyses using filters (0.45 µm, 4 mm nylon filter, Thermo Fisher Scientific, Rockford, USA). HPLC analyses were carried out using an Agilent 1200 liquid chromatography system, equipped with a quaternary solvent delivery system, an autosampler and a photodiode array detector (Agilent, Palo Alto, USA). An Agilent ZORBAX SB-C_18_ column (5 µm, 250×4.5 mm) (Agilent, Palo Alto, USA) was used. The column was kept at 30°C and the flow rate was 1 ml/min. The diode array detector was set at 330 nm. Methanol (MeOH), acetonitrile (MeCN) and H_2_O (0.5% formic acid) were used in the mobile phase. The gradient elution was 0–30 min: 15% MeOH +11.5% MeCN +73.5% H_2_O (0.5% formic acid); 30–40 min: 15% MeOH +15% MeCN +70% H_2_O (0.5% formic acid); 40–55 min: 45% MeCN +55% H_2_O (0.5% formic acid); 55–60 min: 100% MeCN.

### Chemical data analysis

Principal component analysis (PCA) was applied to examine the relative distribution of different populations of *E. breviscapus* according to their chemical concentration data indicated in Table 2. The PCA results could also provide evidence that whether the clusters of populations are discrete and obvious or not. Agglomerative hierarchical clustering (AHC) analysis was applied to construct a dendrogram based on the distance of the population chemical components (Similarity: Pearson correlation coefficient, Agglomeration method: Unweighted pair-group average). All these analyses were carried out using XLSTAT ver. 2011 software (Addinsoft, Paris, France).

**Table 2:** The concentrations of four chemical compounds in 23 *E. breviscapus* populations.

Population ID	Scutellarin (%)	Erigoster B (%)	3,5-Dicaffeoylquinic acid (%)	1,5-Dicafeoylquinic acid (%)
**A**	1.3035	0.2961	0.4018	0.0519
**B**	1.7025	0.1542	0.6486	0.1220
**C**	1.6538	0.1899	0.6531	0.0861
**D**	1.9571	0.5992	0.8886	0.0869
**E**	1.1482	0.2721	0.6567	0.0750
**F**	1.4472	0.3716	0.6258	0.0893
**G**	2.0600	0.7440	1.5320	0.1843
**H**	1.8722	0.0665	0.9654	0.1615
**I**	1.8106	0.4099	0.5231	0.0758
**J**	1.7635	0.3699	0.3912	0.1072
**K**	1.6417	0.2496	0.6795	0.1817
**L**	1.6795	0.2709	1.2431	0.0623
**M**	1.4671	0.2519	0.3452	0.1072
**N**	1.3489	0.4728	0.5563	0.1681
**O**	1.3413	0.4945	0.7091	0.2673
**P**	1.0353	0.2632	0.4115	0.1100
**Q**	1.7080	0.6401	0.5319	0.1619
**R**	1.5004	0.5007	0.4804	0.1707
**S**	2.1946	0.5822	0.7872	0.1688
**T**	2.0684	0.5942	0.4948	0.1259
**U**	1.7462	0.4305	0.5441	0.1184
**V**	1.7994	0.3280	0.5331	0.0866
**W**	0.5804	0.1235	0.2726	0.0951

### AFLP procedure and primer selection

Genomic DNA was extracted from silica-gel-dried leaves using the cetyltrimethylammonium bromide (CTAB) method [30].

The AFLP technique was performed using a slightly modified version of the protocol of Vos et al. [29]. Total genomic DNA (350 ng) was digested with *Eco*RI and *Mse*I restriction enzymes and buffer 4 (New England Biolabs, Beijing, China) at 37°C for 3 h. At 22°C for 3 h, the *Eco*RI and *Mse*I adapters were ligated to the digested DNA fragments subsequently with T4 DNA ligase (New England Biolabs, Beijing, China). PCR pre-amplification was performed using a total volume of 20 µl reaction mixture containing 5 µl of the ligated product, 1.0 µl *Mse*I primer and 1.0 µl *Eco*RI primer (75 ng/µl), 2.0 µl 10×PCR buffer (Mg^2+^ Free, Takara, Kyoto, Japan), 1.6 µl dNTPs (2.5 mM, Tiangen, Beijing, China), 0.2 µl r Taq DNA polymerase (5 U/µl, Takara, Kyoto, Japan), 1.6 µl MgCl_2_ (25 mM, Takara, Kyoto, Japan) and 7.6 µl double deionized water. The pre-amplification cycles started with 5 min denaturation at 65°C, followed by 30 cycles of 30 s at 94°C, 30 s at 56°C, and 1 min at 72°C, and ended with a final extension step at 72°C for 5 min. The subsequent selective-amplification was conducted using a total volume of 20 µl reaction mixture containing 0.6 µl of the pre-amplified product, 1.0 µl *Mse*I selective primer and 1.0 µl *Eco*RI selective primer (75 ng/µl), 2.0 µl 10×PCR buffer (Mg^2+^ Free, Takara, Kyoto, Japan), 1.6 µl dNTPs (2.5 mM, Tiangen, Beijing, China), 0.2 µl r Taq DNA polymerase (5 U/µl, Takara, Kyoto, Japan), 1.6 µl MgCl_2_ (25 mM, Takara, Kyoto, Japan) and 12 µl double deionized water. The selective-amplification cycles began with 2 min denaturation at 94°C, followed by 13 cycles of 30 s at 94°C, 30 s at 65°C (decreasing by 0.7°C per cycle), and 1 min at 72°C, subsequently accompanied by 23 cycles of 30 s at 94°C, 30 s at 56°C, and 1 min at 72°C, and ended with a final extension step at 72°C for 5 min.

A total of 23 primer combinations, which were previously used to study taxa in Asteraceae [31]–[33], were tested using 5 populations of the DNA samples, and the following three primer pairs were then chosen for the further analyses: *Eco*RI + ACC/*Mse*I + CAG, *Eco*RI + ACG/*Mse*I + CAG, *Eco*RI + ACA/*Mse*I + CAC, labeled with FAM. The fragment analyses were performed with ABI PRISM 3730xl Avant Genetic Analyzer (Applied Biosystems, Foster City, USA).

### AFLP scoring

The fragments, 60–450 base pairs (bp) in length, were scored as present (1) or absent (0) in the software Genemapper 4.0 (Applied Biosystems, Foster City, USA). For all of the primer pairs, the selection of markers for scoring involved two steps. First, the peaks which exceeded 300 relative fluorescent units (RFU) were coded as present, while ones below this criterion were considered as absent. Second, the AFLP binary matrices of these primer pairs were checked for any mistakes or ambiguities by comparing with the original files of graphs, and corrected for further analysis. Poor amplifications occurred only with 4 individuals, two from population B, one from population O, one from population U, and these samples were excluded from the further analysis in order to reduce artificial bias. For further data analyses, the original AFLP data matrix was converted into an input file for ARLEQUIN software using AFLPDAT [34], a collection of R functions [35].

### Genetic data analysis

To estimate genetic variation, POPGENE ver. 1.31 [36] was used to calculate the percentage of polymorphic bands (PPB), and the total genotypic diversity (*H*_T_), the genotypic diversity within populations (*H*_S_), based on Nei's [37] measures of gene diversity. The coefficient of gene differentiation (*G*_ST_) was calculated as follows: *G*_ST_ = (*H*_T_ - *H*_S_)/*H*_T_, in POPGENE ver. 1.31. The effective number of migrants per generation, an indirect estimate of the gene flow between any pair-wise populations, was estimated as: *Nm* = 0.5 (1 - *G*_ST_)/*G*_ST_ [38]. The reason why the value 0.5 was substituted for 0.25 in the formula is that the AFLP marker is a dominant maker which is similar to haplotypic data. Furthermore, the partitioning of variation at different levels was calculated by Analysis of Molecular Variance (AMOVA) [39] in ARLEQUIN ver. 3.11 [40] using 10,000 permutations.

The unweighted pair group method with averages (UPGMA) clustering analysis, based on the Nei's unbiased genetic distance [41], was calculated using the TFPGA ver. 1.3 [42], and the bootstrap values were derived from 10,000 permutations.

### Combined data analysis

To eliminate the effects of different scales of measurement, the chemical data were standardized using the Standardization Module in NTSYSPC ver. 2.11c software [43]. Subsequently, the chemical distance was derived from the standardized matrix gained from the previous calculation using the Interval Data Module and Euclidean Distances Method in NTSYSPC ver. 2.11c software. Then, this triangle matrix was used as one matrix in the input file for Mantel Test [44]. The population genetic pairwise distance (*F*_ST_) matrix was calculated as the other matrix. Then, the relationship between the population genetic pairwise distance (*F*_ST_) matrix and the population chemical pairwise distance matrix was computed using a Mantel test with 10,000 permutations in ARLEQUIN ver. 3.11. We used the same methods mentioned above to calculate the relationship between chemical pairwise distance and geographic distance (calculated using latitudes and longitudes of sampling locations). We also tested the relationship between the population genetic pairwise distance (*F*_ST_) and the geographic distance (calculated using latitudes and longitudes of sampling locations) using Mantel test.

The concentrations of the four chemical components measured by HPLC and the environmental data gathered from meteorological stations and field work were combined together and analyzed by correlation test in XLSTAT ver. 2011 software, in order to detect the correlation among them.

## Results and Discussion

### Chemical components and analysis

**Figure 2:**
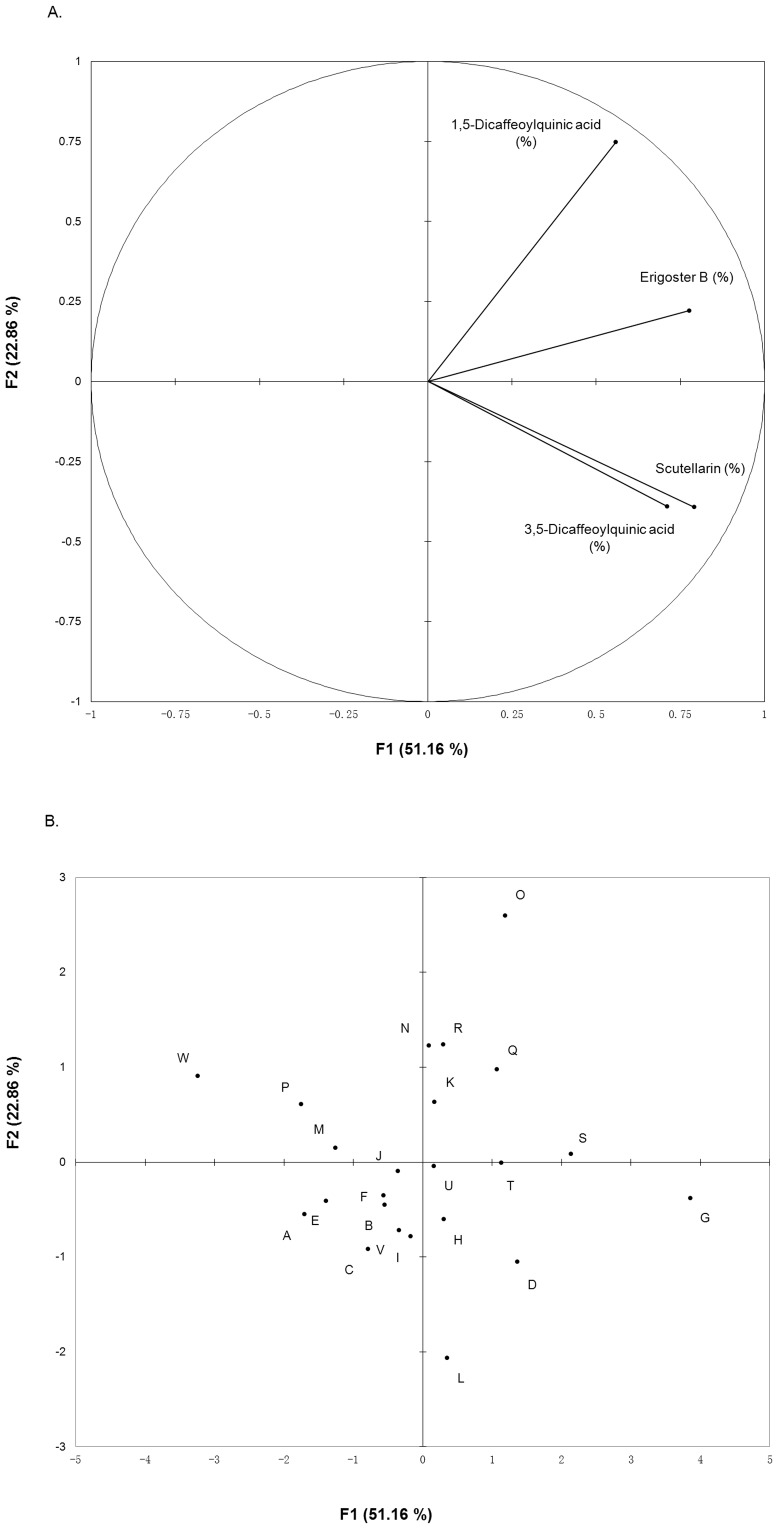
PCA of four chemical components of *E. breviscapus* including (A) the PCA distribution of variables and (B) the PCA distribution of samples (capital letters denote populations).

**Figure 3:**
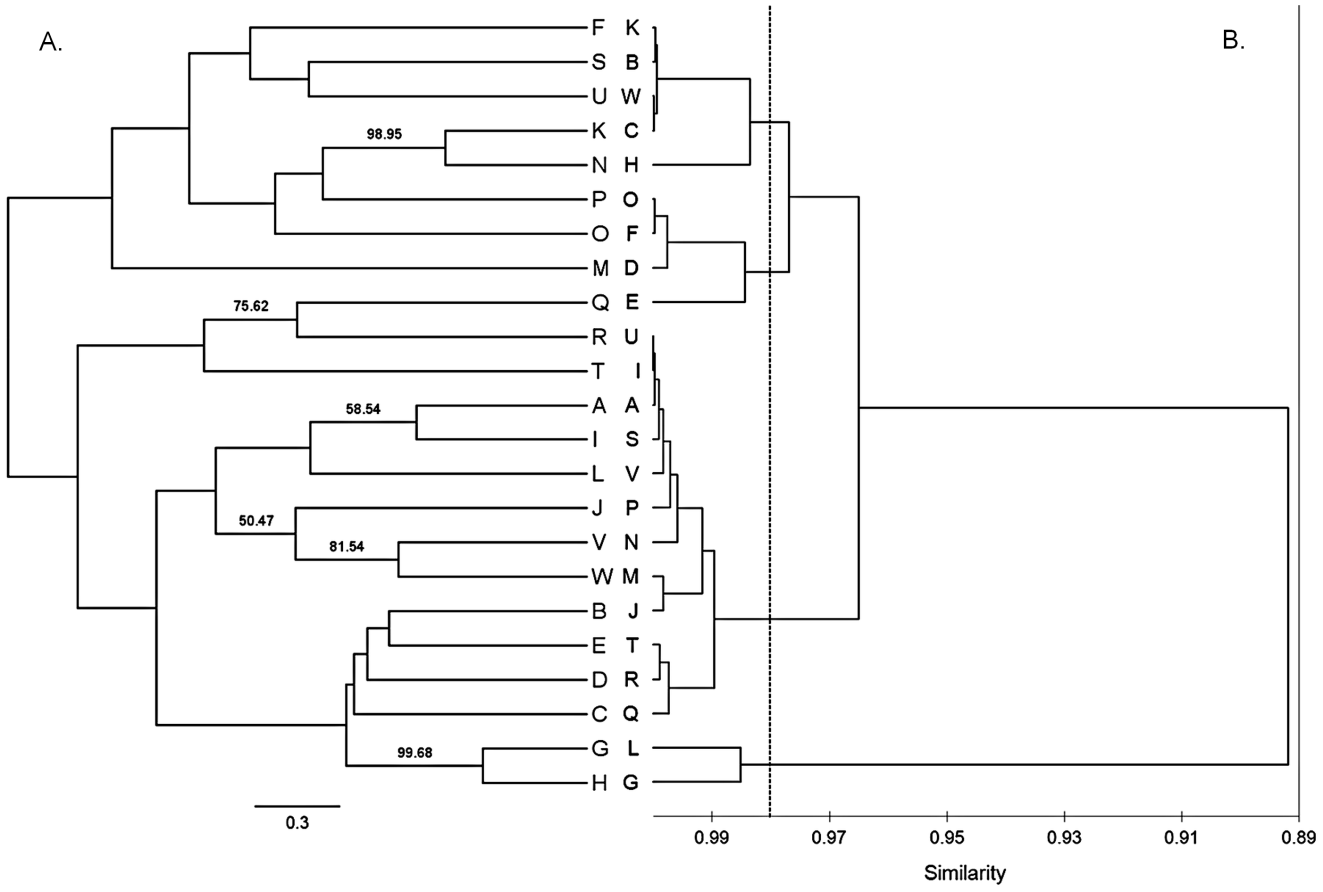
Comparison between genetic dendrogram. (**A**) the UPGMA dendrogram with bootstrap values (>50%) derived from unbiased genetic distance (left) and (**B**) chemical dendrogram, the dendrogram of 23 *E. breviscapus* populations derived from the Pearson correlation coefficient of four chemical compounds (right) (capital letters denote the populations).

The concentration of scutellarin varied from 0.5804% to 2.1946% among 23 populations, erigoster B ranged from 0.0665% to 0.6401%, 3,5-dicaffeoylquinic acid ranged from 0.2726% to 1.5320% and 1,5-dicaffeoylquinic acid varied from 0.0519% to 0.2673% (Table 2). Based on the chemical characteristics, the PCA distribution plot of samples and variables were displayed in Figure 2, and the eigenvector values were listed in Table 3. The dendrogram of AHC was presented in Figure 3B.

**Table 3:** The eigenvector values obtained from the PCA analyses on four chemical components of *E. breviscapus*.

	F1	F2	F3	F4
**Scutellarin (%)**	0.553	−0.411	−0.266	−0.674
**Erigoster B (%)**	0.543	0.232	−0.599	0.541
**3,5-Dicaffeoylquinic acid (%)**	0.497	−0.409	0.653	0.400
**1,5-Dicaffeoylquinic acid (%)**	0.390	0.781	0.379	−0.306

The PCA distribution plot of samples suggested that the chemical characteristics differentiated populations from each other, but no obvious cluster was detected among the 23 populations (Fig. 2B). Interestingly, the PCA distribution of variables revealed a positive relationship between the contents of scutellarin and 3,5-dicaffeoylquinic acid (Fig. 2A). The dendrogram (Fig. 3B) of chemical characteristics divided the 23 populations into four clades: Clade I (population B, C, H, K and W), Clade II (population D, E, F and O), Clade III (population A, I, J, M, N, P, Q, R, S, T, U and V) and Clade IV (population G and L). The results of PCA and AHC suggested that the 23 populations could be divided into four clades, but this clustering pattern was vague, i.e. the chemical variation of the 23 populations was somewhat continuous. Furthermore, to consider biogeographic connections, we compared the four clades calculated according to the four chemical components with the sampling map (Fig. 1). The clades had no obvious correlations with the geographic distribution, i.e. populations sharing more similar chemical diversification do not necessarily mean they have closer distribution. Uncorrelation between the chemical pairwise distance and geographic distance was revealed by the Mantel test (*r* = 0.068, *P* = 0.235), which further supported this conclusion.

### Genetic data analysis based on AFLP data

A total of 521 AFLP fragments were obtained for the three primer combinations used, with 504 (PPB = 96.74%) polymorphic bands. With the assumption of Hardy-Weinberg equilibrium, the total Nei's genetic diversity [37] of the species (*H*_T_) was 0.134, while the average Nei's genetic diversity within population (*H*_S_) was 0.102. Analysis of Molecular Variance (AMOVA) based on all populations as one group indicated that majority of genetic variation (76.78%; *P* = 0.000) occurred within populations, while the variation among populations was 23.22% (*P* = 0.000). The coefficient of gene differentiation (*G*_ST_) was 0.241, which was similar to the result obtained with the AMOVA. These results indicated more genetic variation was within populations, which suggested the differentiation among populations was not significant.

The result of *Nm* suggested a high level of inter-population gene flow (*Nm* = 1.575), which means there were 1.575 migrants per generation. According to Wright [45], if *Nm* <1, local populations tend to differentiate, whereas there will be little differentiation among populations and migration is more important than genetic drift, if *Nm* ≥1. The high value of *Nm* in this species indicated that gene flow among populations is quite frequent, which caused low intraspecific differentiations.

To further detect the population structure and find potential clusters, the UPGMA tree was calculated and the tree with bootstrap values (>50%) is shown in Figure 3A. As depicted in the dendrogram, all the basal branches and most terminal branches were poorly supported (<50%), which indicated no obvious cluster was detected and the differentiation among populations was not significant. However, populations (e.g., G and H; K and N; Q and R; V and W) clustered in well supported clades and have close locations. Furthermore, the Mantel test also suggested correlations between the genetic pairwise distance (*F*_ST_) and geographic distance (*r* = 0.485; *P*<0.01), i.e. the genetically close populations are distributed close to one another.

### Combined data analysis

The relationship between genetic differentiation and chemical differentiation

The comparison of the genetic UPGMA tree and the dendrogram of chemical characteristics (Fig. 3), which produced by same clustering methods, revealed no obvious correlation between genetic differentiation and chemical differentiation. The populations belonging to four clades in the chemical dendrogram were almost randomly distributed through the genetic UPGMA tree. Furthermore, the Mantel test of the relationship between the population genetic pairwise distance (*F*_ST_) and chemical pairwise distance (*r* = −0.0497; *P* = 0.6636) showed that population genetic differentiation did not correlate with the concentration of the chemical components. However, the molecular markers indicated weak genetic differentiation among *E. breviscapus* populations. Meanwhile, the PCA plot suggested no obvious clusters based on the four chemical components, which indicated that the chemical differentiation among populations had no clear clustering pattern (Fig. 2). The insignificant genetic differentiation and continuous chemical differentiation might weaken the correlation between them. The weak genetic differentiation among populations may cause the indistinct chemical clustering pattern. The low genetic differentiation may be attributable to its outcrossing mating system [4] and tiny wind-dispersed fruits, which caused high gene flow among populations. Furthermore, pooling samples according to population without considering the intra-population differentiation may also result in the insignificant correlation mentioned above.

### The relationship between the environmental factors and chemical differentiation

Because of the missing meteorological data (Table 1), four populations (E, G, S and U) were omitted in the following analyses related to meteorological data.

According to correlation test results (Table 4 and 5), there was a positive relationship between the concentration of 1,5-dicaffeoylquinic acid and latitude. However, the correlation between the content of 1,5-dicaffeoylquinic acid and annual average temperature was negative. These two results are not paradoxical, since high latitude largely correspond to a lower temperature across the distribution of *E. breviscapus*. 1,5-dicaffeoylquinic acid may have functions in adapting to relatively low temperature in *E. breviscapus*. It is the first time that positive correlations between the concentrations of phenols in *E. breviscapus* and environmental temperature as well as latitude is detected, which could be used to improve the content of related phenols by choosing a cooler area to cultivate *E. breviscapus*. Within the natural distribution of *E. breviscapus*, relatively higher locations will be more ideal for cultivating *E. breviscapus*, especially to generate higher concentration of 1,5-dicaffeoylquinic acid.

**Table 4:** Results of correlation test based on 23 *E. breviscapus* populations (without meteorological data).

Variables	Scutellarin (%)	Erigoster B (%)	3,5-Dicaffeoylquinic acid (%)	1,5-Dicafeoylquinic acid (%)	Altitude	Latitude	Longitude
**Scutellarin (%)**	****	**0.016**	**0.015**	0.467	0.968	0.948	0.472
**Erigoster B (%)**	**0.495**	****	0.181	0.069	0.617	0.078	0.947
**3,5-Dicaffeoylquinic acid (%)**	**0.500**	0.289	****	0.309	0.570	0.661	0.414
**1,5-Dicafeoylquinic acid (%)**	0.160	0.386	0.222	****	0.185	**0.012**	0.117
**Altitude**	−0.009	−0.110	−0.125	0.287	****	0.952	**0.044**
**Latitude**	−0.015	0.375	−0.097	**0.515**	0.013	****	0.202
**Longitude**	0.158	0.015	0.179	−0.336	**−0.424**	−0.276	****

Values in bold are different from 0 with a significant level, alpha = 0.05.

Correlation matrix (Pearson) is listed below the diagonal, while the *P*-values are listed above the diagonal.

**Table 5:** Results of correlation test between chemical compounds and meteorological data in 19 *E. breviscapus* populations (excluding population E, G, S and U).

Variables	Scutellarin (%)	Erigoster B (%)	3,5-Dicaffeoylquinic acid (%)	1,5-Dicafeoylquinic acid (%)
**Annual average temperature (°C)**	0.370 (0.119)	−0.013 (0.958)	0.087 (0.724)	**−0.690 (0.001)**
**Annual average precipitation (mm)**	0.419 (0.074)	−0.267 (0.269)	**0.495 (0.031)**	0.065 (0.790)
**Annual average hours of sunshine (h)**	−0.164 (0.501)	0.187 (0.444)	−0.290 (0.229)	0.032 (0.895)

Values in bold are different from 0 with a significant level, alpha = 0.05.

*P*-values are listed in parentheses.

The concentration of 3,5-dicaffeoylquinic acid was positively correlated with annual average precipitation, which means that a moist environment may enhance the synthesis of 3,5-dicaffeoylquinic acid. This result is consistent with the research on the effect of water stress on *E. breviscapus*, which suggested waterlogging and drought can raise the content of phenols in *E. breviscapus* [22]. Hence, soil moisture can be increased to augment the yield of 3,5-dicaffeoylquinic acid.

As to the concentrations of scutellarin and erigoster B, they had no relationship with any of the environmental factors listed in Table 1. Therefore, according to this study, the cultivation modifications mentioned above should not reduce the concentrations of scutellarin and erigoster B.

### The relationship among the three chemical compounds

**Figure 4:**
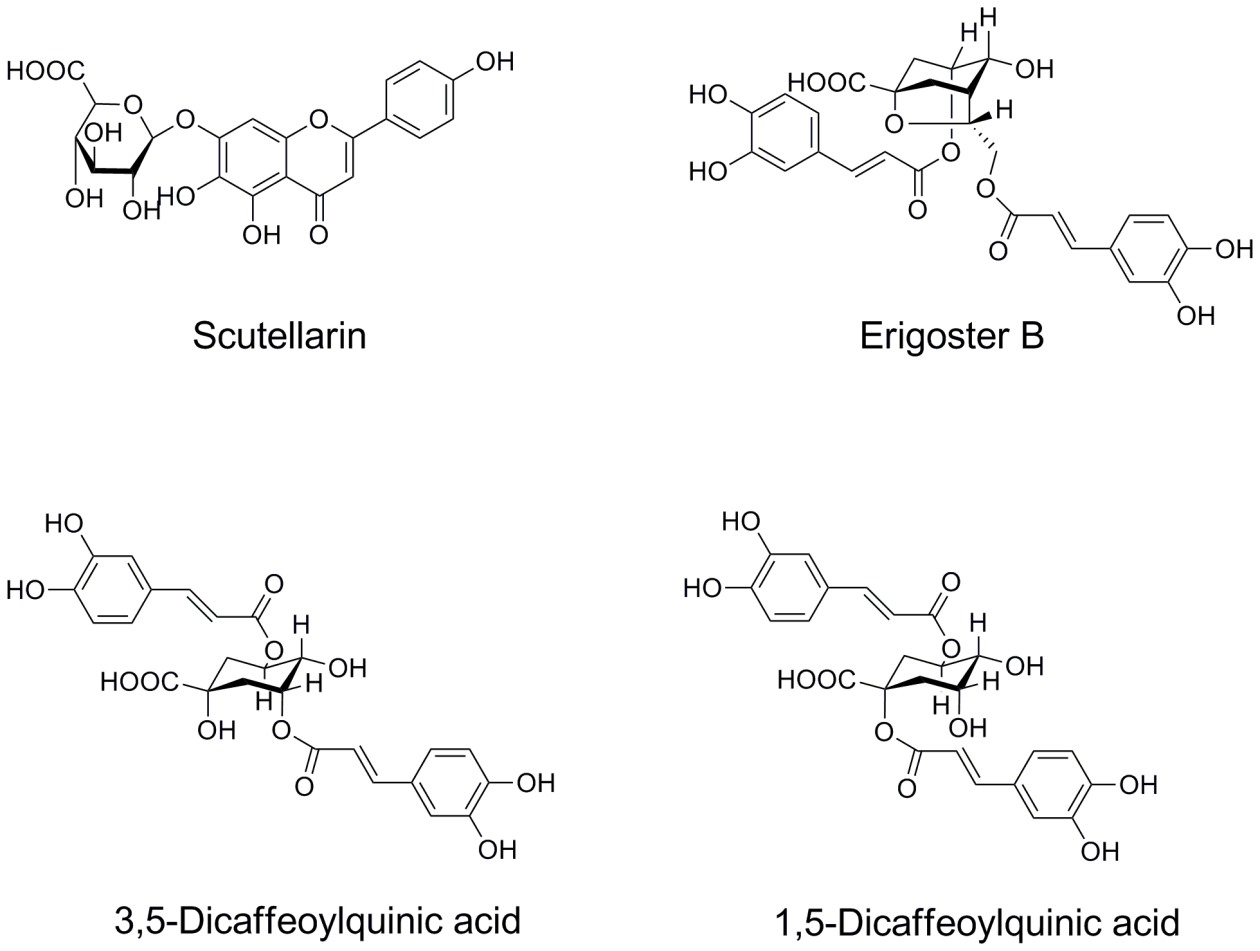
Chemical structures of scutellarin, erigoster B, 1,5-dicaffeoylquinic acid and 3,5-dicaffeoylquinic acid.

Interestingly, the correlation test revealed a positive relationship between two pairs of the four chemical compounds investigated. The concentration of scutellarin was inferred to be positively related to concentrations of erigoster B and 3,5-dicaffeoylquinic acid, respectively. As discussed in the introduction, scutellarin is a flavonoid, whereas erigoster B and 3,5-dicaffeoylquinic acid are part of the conjugated hydroxycinnamate class, and their chemical structures are shown in Figure 4. Both erigoster B and 3,5-dicaffeoylquinic acid contain a molecule of caffeic acid and a molecule of 5-*O*-caffeoylquinic acid. These phenols are produced via the shikimate pathway and phenylpropanoid metabolism [19]. In phenylpropanoid metabolism, phenylalanine is initially converted to cinnamic acid by the action of phenylalanine ammonia-lyase (PAL). Finally, cinnamic acid is converted to 4-coumaroyl-CoA, which will be further converted into flavonoids through the flavonoid pathway or hydroxycinnamates by another pathway [46]. According to Crozier et al. [47], the synthesis of flavonoids and chlorogenic acid (5-*O*-caffeoylquinic acid, the precursor of hydroxycinnamate) diverges from the 4-coumaroyl-CoA. Therefore, according to the positive relationship between the scutellarin and erigoster B as well as 3,5-dicaffeoylquinic acid, we hypothesize that these two pairs of compounds may share the same stimulus-triggering mechanism that enhance the production of the reactions in the shikimate pathway and phenylpropanoid metabolism, i.e. these compounds may have similar ecological adaptation functions in *E. breviscapus*.

## Conclusions

In sum, low genetic and successive chemical differentiations among populations of *E. breviscapus* were detected, and the genetic differentiation was largely unrelated to the chemical differentiation. It is thus hard to select high quality germplasm resources according to genetic lineage. Appropriate cultivation conditions should be applied to increase the yields of related compounds efficiently. The concentrations of 3,5-dicaffeoylquinic acid and 1,5-dicaffeoylquinic acid could be increased through decreasing the cultivation temperature and increasing the moisture. The climate of high latitude regions can enhance the yield of 1,5-dicaffeoylquinic acid significantly, and thus it can be considered a preferred cultivation areas for *E. breviscapus*. Furthermore, the positive correlation between the concentration of scutellarin and that of erigoster B as well as 3,5-dicaffeoylquinic acid maybe attributable to the same stimulus that induces the shared parts of their synthesis pathway, whose mechanisms need more thorough examination.
